# Sensorless Contact Force Estimation and Adaptive Variable-Damping Compliant Control for Biomimetic Robotic Arm

**DOI:** 10.3390/biomimetics11090637

**Published:** 2026-09-05

**Authors:** Yanwei Xie, Jiawen He, Yi Zhang

**Affiliations:** 1 State Key Laboratory of Precision Manufacturing for Extreme Service Performance, Central South University, Changsha 410083, China; xieyanwei@csu.edu.cn; 2 College of Mechanical & Electrical Engineering, Central South University, Changsha 410083, China; 3 School of Artificial Intelligence and Robotic, Hunan University, Changsha 410082, China; hjwdaxia@hnu.edu.cn

**Keywords:** strong tracking Kalman filter (SKF), contact force, fuzzy control, adaptive control, impedance control

## Abstract

To address contact force estimation and compliant control for biomimetic robotic arms interacting with uncertain environments, an adaptive variable-damping impedance control method based on a fuzzy-controlled forgetting-factor strong tracking Kalman filter (FSKF) is proposed. The proposed method improves the conventional strong tracking Kalman filter (SKF) by introducing a fuzzy control strategy to adaptively adjust the forgetting factor, thereby enhancing the filtering performance and improving the accuracy of contact force estimation. The estimated contact force is subsequently incorporated into an adaptive variable-damping impedance controller to achieve simultaneous contact force estimation and compliant control of the biomimetic robotic arm. During biomimetic robotic arm motion, the proposed controller utilizes the estimated contact force to adaptively regulate the damping coefficient, compensating for force-tracking errors caused by environmental uncertainties and thereby improving both force and position tracking performance. The simulation and experimental results demonstrate that the proposed adaptive variable-damping impedance controller has better force and position tracking accuracy compared with the conventional impedance controller. Compared with traditional methods, the estimation accuracy based on FSKF has improved by about 7.3%. These results demonstrate the potential of the proposed method for prosthetic systems and other applications involving compliant robot–environment interaction.

## 1. Introduction

Biomimetic robotic arms are increasingly used in prosthetic and rehabilitation devices, collaborative manipulation, and other applications involving physical interaction with uncertain environments [1,2,3]. These systems must achieve accurate position tracking while regulating contact forces safely. However, unknown environmental stiffness, time-varying contact conditions, and measurement noise may cause excessive forces or tracking errors when pure position control is used [4,5]. Therefore, sensorless contact force estimation combined with compliant control is important for robotic systems.

Contact forces can be measured directly using force/torque sensors or estimated from joint states, motor torques, and dynamic models. Although sensor-based methods provide direct measurements, dedicated sensors increase system mass, cost, wiring complexity, and integration requirements [6,7,8]. Model-based sensorless methods avoid these disadvantages and commonly employ Kalman filtering, time-delay estimation, or model compensation [9]. Extended and unscented Kalman filters have been used for external-force estimation in robotic manipulators and prosthetic systems, while adaptive filtering and motor-current-based virtual sensing have also been investigated. Jung et al. used a Lyapunov adaptive law to correct the input state and estimated the contact force of a 3-DOF robotic arm using an extended Kalman filter [10]. Fakoorian et al. used external angle and angular velocity sensors and applied an unscented Kalman filter to estimate the contact force at the end of a 4-DOF foot [11]. Roveda et al. used an extended Kalman filter to estimate the torque of a 7-DOF robot [12]. Duan et al. adjusted the damping term online according to the accumulated force error, compensated for system error, and achieved force and position tracking [13]. Cao et al. improved the Kalman filter using a variable-gain adaptive moving-average technique, allowing the system to automatically switch the filter gain according to real-time feedback and successfully estimate robot contact force [14]. Wahrburg et al. used an improved Kalman filter to convert low-level motor current, joint position, and velocity information of a robotic arm into a virtual force sensor to estimate external contact forces and torques in Cartesian space [15]. However, their performance may be affected by dynamic-model uncertainty, friction, noisy motor signals, manually selected parameters, and abrupt contact force changes.

Data-driven and nonlinear-observer methods provide alternative solutions under model uncertainty. Neural-network-based approaches can learn complex relationships between robot states and interaction forces, whereas finite-time observers can provide rapid estimation. Wu et al. combined convolutional neural networks, gated recurrent units, and force transformation in a data-driven algorithm to estimate robot end-effector contact force without an accurate physical model [16]. Lin et al. constructed a force observer using a temporal neural network based on the Transformer architecture and achieved estimation of robot end-effector contact force under strong model uncertainty [17]. Han et al. used a high-order finite-time observer based on nonlinear system theory to rapidly estimate contact force under model uncertainty [18]. Nevertheless, data-driven methods require representative training data and may involve considerable computational cost. Nonlinear observers often depend on prior assumptions about disturbance bounds and carefully tuned gains. Thus, existing sensorless estimation methods involve a trade-off among hardware complexity, model dependence, transient response, computational cost, and robustness.

Among these approaches, model-based Kalman filtering is particularly attractive for robotic contact-force estimation because it can recursively fuse joint-state and motor-torque measurements without requiring additional force sensors, while maintaining relatively low computational complexity [9,10,11,12,13,14,15]. This makes it suitable for lightweight robotic arms operating in real time. However, the transition from free space to contact introduces abrupt changes in the estimated state and exposes the filter to model mismatch and measurement noise. Consequently, the adaptability of the filter parameters is critical for maintaining accurate and timely contact-force estimation. Strong tracking Kalman filters (SKF) can improve the response of conventional Kalman filters under model mismatch and sudden state changes by introducing a forgetting factor. However, this factor is commonly selected using prior experience or offline simulations, which limits the adaptability of the filter under time-varying conditions. Fuzzy logic has been introduced to adapt the parameters of strong tracking filters. Zhang et al. proposed a strong tracking Kalman filter with a fuzzy forgetting factor [19], while Jwo et al. developed a fuzzy strong tracking unscented Kalman filter for integrated navigation [20]. These studies demonstrate the potential of fuzzy adaptation for improving filter performance. However, the application of this mechanism to sensorless contact force estimation in high-degree-of-freedom biomimetic robotic arms, and its subsequent use in force-feedback impedance control, has not been sufficiently investigated.

Impedance control establishes a dynamic relationship between position error and interaction force. Conventional fixed-parameter impedance controllers are simple and generally stable, but they cannot provide an optimal balance between tracking accuracy and compliance when environmental stiffness changes [21]. Adaptive and variable-impedance methods adjust virtual parameters according to the motion state, force error, or estimated environmental characteristics. Zahedi et al. used a Bayesian optimization algorithm to adaptively adjust the sign and magnitude of damping according to the user’s motion intention, significantly improving the agility of human–robot interaction and reducing energy consumption [3]. Sun et al. designed a variable impedance control law for robots with modeling uncertainties using an approximate dynamic inversion method combined with the extended Tikhonov theorem, effectively improving the compliance of robots in dynamic interaction [22]. Xiong et al. proposed an arm-angle-based null-space impedance control strategy, realizing compliant control of a 7-DOF robotic arm and ensuring environmental compliance of the robotic elbow [23]. Rhee et al. proposed a hybrid impedance and admittance control strategy for robotic arms in unknown environments. By using an adaptive algorithm to identify environmental parameters online, the strategy can achieve compliant control and accurate torque tracking when the contact position and environment are unknown [24]. However, existing methods may depend on environmental assumptions, online parameter convergence, or optimization procedures. In addition, the combined influence of noisy force estimates, abrupt contact transitions, and time-varying environmental stiffness remains insufficiently addressed.

Based on the above analysis, three main challenges remain. First, external force sensors increase the mass and cost of robotic systems. Second, conventional filtering methods may have limited transient adaptability because their parameters are fixed or manually tuned. Third, fixed-parameter impedance control may not maintain accurate force and trajectory tracking under changing environmental conditions. In this study, the fuzzy strong tracking Kalman filter (FSKF) addresses the second challenge by adjusting the forgetting factor according to the state-variable estimation error. The adaptive variable-damping impedance controller addresses the third challenge by adjusting the damping coefficient according to the force-tracking error. Together, they provide a sensorless force-feedback framework that reduces the dependence on dedicated force sensors.

Accordingly, this study proposes an FSKF-based contact force estimator integrated with an adaptive variable-damping impedance controller for a 7-DOF biomimetic robotic arm. The main contributions are as follows:

1. A fuzzy strong tracking Kalman filter is developed to improve the transient response of sensorless contact force estimation during abrupt contact.
2. An adaptive variable-damping impedance controller is designed to compensate for force-tracking errors caused by changes in environmental stiffness.
3. The integrated framework is evaluated through simulations and experiments involving contact force estimation, force tracking, and trajectory tracking. The prosthetic-arm prototype is used as a representative biomimetic robotic-arm platform, while the study focuses on algorithmic validation rather than complete human-in-the-loop prosthetic operation.

## 2. Model Description

**Figure 1:**
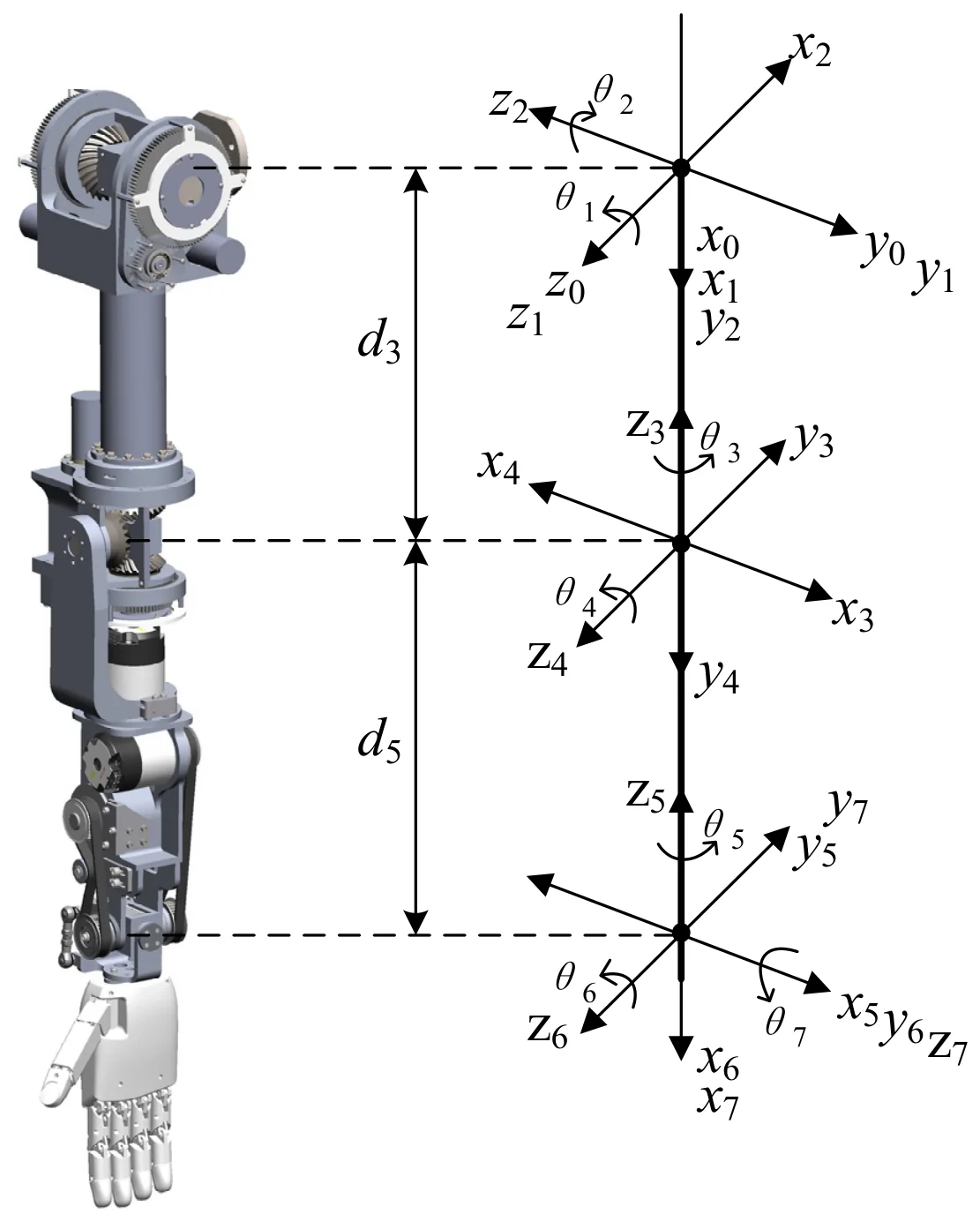
Biomimetic robotic arm model and DH coordinate frames.

A 7-DOF biomimetic robotic arm provides an anthropomorphic structure and sufficient motion redundancy, making it a representative platform for studying contact force estimation and compliant control. Such a platform can be used in prosthetic devices, rehabilitation systems, and other human-centered robotic applications. The structure of the 7-DOF upper-limb biomimetic robotic arm is shown in Figure 1. The model consists of the upper arm, forearm, and hand. The shoulder joint controls abduction/adduction, flexion/extension, and circumduction of the upper arm; the elbow joint controls flexion/extension and rotation of the forearm; and the wrist joint enables flexion and deviation of the hand. According to [25], the DH parameters are listed in Table 1.

According to [26], the dynamics of the robotic arm are established using the Euler–Lagrange equation:

(1) $$\left\{\begin{array}{c} \frac{d}{dt}{\left(\frac{\partial L}{\partial \dot{q}}\right)}^{T}-{\left(\frac{\partial L}{\partial q}\right)}^{T}=\tau \\ L=T-U \end{array}\right.$$

where L is the Lagrange equation, T is the kinetic energy of the system, U is the potential energy of the system, and τ is the generalized torque/force.

Considering external and friction torques, the dynamic equation of the 7-DOF biomimetic robotic arm can be obtained from Equation (1) as follows:

(2) $$B\left(q\right)\ddot{q}+C\left(q,\dot{q}\right)\dot{q}+g\left(q\right)+\tau_{f}\left(\dot{q}\right)=\tau -\tau_{ext}$$

where B(q) is the inertia matrix, $C(q,\dot{q})$ is the centrifugal and Coriolis matrix, g(q) is the gravity matrix, $\tau_{f}\left(\dot{q}\right)$ is the joint friction matrix, τ is the torque input matrix, and $\tau_{ext}$ is the external torque vector generated after the robotic arm contacts the environment.

The state variable of the robotic arm is $x={\left[\begin{array}{cc} q & \dot{q} \end{array}\right]}^{T}$, and its state equation is as follows:

(3) $$\dot{x}=\left[\begin{array}{c} \dot{q}\\ \ddot{q} \end{array}\right]=\left[\begin{array}{c} \dot{q}\\ B{\left(q\right)}^{-1}\left(\tau -C(q,\dot{q})\dot{q}-g(q)-\tau_{f}(\dot{q})-\tau_{ext}\right) \end{array}\right]$$

## 3. Contact Force Estimation Based on the FSKF

For robotic systems that require low mass, low cost, and compact integration, including prosthetic and wearable platforms, installing dedicated contact force sensors may be undesirable [6]. Meanwhile, to obtain interaction information between the robotic arm and the external environment, such as contact force, an algorithm is needed that can obtain contact force estimates and other interaction information in real time from existing information. The Kalman Filter (KF) is a linear filter with the ability to fuse multivariate uncertain information for optimal state estimation. Its use depends on system linearity and Gaussian-distributed noise, and the filter consists of a prediction equation and a measurement equation [27].

### 3.1. Strong Tracking Kalman Filter

The extended Kalman filter (EKF) is a nonlinear form of the KF and estimates the dynamic state of a nonlinear system through local linearization [27]. To estimate the external torque, the external torque ($\tau_{\mathrm{ext}}$) is augmented into the state vector; therefore, the state vector becomes $x={\left[q,\dot{q},\tau_{ext}\right]}^{T}$. Substituting the augmented state variable into Equation (3), and considering that $\tau_{ext}$ is generally constant or slowly varying, it is assumed that ${\dot{\tau }}_{ext}=0$ [6,28]. The new state equation of the robotic arm is

(4) $$\dot{x}=\left[\begin{array}{c} \dot{q}\\ \ddot{q}\\ {\dot{\tau }}_{ext} \end{array}\right]=\left[\begin{array}{c} \dot{q}\\ B{\left(q\right)}^{-1}\left(\tau -C(q,\dot{q})\dot{q}-g(q)-\tau_{f}(\dot{q})-\tau_{ext}\right)\\ 0 \end{array}\right]=f\left(x,\tau \right)$$

The observation equation of the robotic arm is

(5) $$y=Hx=\left[\begin{array}{ccc} I_{7\times 7} & 0 & 0\\ 0 & I_{7\times 7} & 0 \end{array}\right]\left[\begin{array}{c} q\\ \dot{q}\\ \tau_{ext} \end{array}\right]$$

Considering uncertainties in the system and measurements, process noise w and measurement noise v are introduced into Equations (4) and (5), and the state-space model can be written as

(6) $$\left\{\begin{array}{c} \dot{x}=f\left(x,\tau \right)+w\\ y=Hx+v \end{array}\right.$$

Discretizing Equation (6) with the sampling time $T_{s}$, the state-space model becomes

(7) $$\left\{\begin{array}{c} x_{k+1}=x_{k}+T_{s}\cdot f\left(x_{k},\tau_{k}\right)+w_{k}\\ y_{k}=Hx_{k}+v_{k} \end{array}\right.$$

where

$$f\left(x_{k},\tau_{k}\right)=\left[\begin{array}{c} f_{1}\\ f_{2}\\ f_{3} \end{array}\right]=\left[\begin{array}{c} {\dot{q}}_{k}\\ B{\left(q_{k}\right)}^{-1}\left(\tau_{k}-C(q_{k},{\dot{q}}_{k}){\dot{q}}_{k}-g(q_{k})-\tau_{f}({\dot{q}}_{k})-{\hat{\tau }}_{extk}\right)\\ 0 \end{array}\right]$$

The SKF is obtained by modifying the EKF algorithm. The EKF algorithm mainly has two shortcomings: (1) when model uncertainty is strong, the estimated state may become inaccurate or even diverge; and (2) when the system reaches a steady state, the algorithm loses its ability to track abrupt state changes [29]. The SKF algorithm uses the orthogonality principle and introduces a fading factor to dynamically adjust the Kalman gain K, thereby effectively compensating for the limitations of the EKF algorithm. In the SKF algorithm, a fading factor matrix $\lambda_{k}=\mathrm{diag}\left[\begin{array}{cccc} \lambda_{1,k} & \lambda_{2,k} & \ldots & \lambda_{i,k} \end{array}\right]$ is introduced into the EKF structure. Based on prior experience, the initial fading factor matrix $\lambda =\alpha =\mathrm{diag}\left[\begin{array}{cccc} \alpha_{1} & \alpha_{2} & \ldots & \alpha_{i} \end{array}\right]$ is determined, where $\alpha_{i}\geq 1$.

Time update:

(8) $${\hat{x}}_{k}^{-}={\hat{x}}_{k-1}+T_{s}\cdot f\left({\hat{x}}_{k-1},u_{k-1}\right)$$

(9) $$P_{k}^{-}=\lambda_{k}F_{k-1}P_{k-1}F_{k-1}^{T}+Q_{k-1}$$

Measurement update:

(10) $$K_{k}=P_{k}^{-}H_{k}^{T}{\left(H_{k}P_{k}^{-}H_{k}^{T}+R_{k}\right)}^{-1}$$

(11) $${\hat{x}}_{k}={\hat{x}}_{k}^{-}+K_{k}\left(y_{k}-H_{k}{\hat{x}}_{k}^{-}\right)$$

(12) $$P_{k}=\left(I-K_{k}H_{k}\right)P_{k}^{-}$$

where

$w_{k}$ and $v_{k}$ are the process noise and measurement noise, respectively, $Q_{k}$ and $R_{k}$ are the process noise covariance matrix and measurement noise covariance matrix, respectively;
$F_{k}$ is the Jacobian matrix of f, and $H_{k}$ is the Jacobian matrix of h;
${\hat{x}}_{k}^{-}$ is the prior estimate, and $P_{k}^{-}$ is the prior error covariance matrix;
${\hat{x}}_{k}$ is the posterior estimate, and $P_{k}$ is the posterior error covariance matrix;
$K_{k}$ is the Kalman filter gain matrix.

The fading factor matrix $\lambda_{k}$ can be obtained iteratively by the following algorithm:

(13) $$\gamma_{k}=y_{k}-H_{k}x_{k}^{-}$$

(14) $$\lambda_{i,k}=\left\{\begin{array}{c} \alpha_{i}c_{k}\;,\;\alpha_{i}c_{k}\geq 1\\ 1\;,\;\alpha_{i}c_{k}<1 \end{array}\right.$$

(15) $$c_{k}=tr\left(N_{k}\right)/tr\left(M_{k}\right)$$

(16) $$N_{k}=V_{k}-H_{k}Q_{k}H_{k}^{T}-\beta R_{k}$$

(17) $$M_{k}=H_{k}F_{k}P_{k-1}F_{k}^{T}H_{k}^{T}$$

(18) $$V_{k}=\left\{\begin{array}{cc} \gamma_{1}\gamma_{1}^{T} & k=1\\ \frac{\rho V_{k-1}+\gamma_{k}\gamma_{k}^{T}}{1+\rho } & k>1 \end{array}\right.$$

where $\gamma_{k}$ is the measurement residual, namely the estimation error; $c_{k}$ is an undetermined factor; β is the weakening factor, with β≥1; ρ is the forgetting factor, with 0<ρ≤1; and $V_{k}$ is the error variance matrix.

It can be seen from the SKF algorithm that, when the state changes, the estimation error $\gamma_{k}$ increases and the error variance matrix $V_{k}$ increases, thereby increasing the fading factor $\lambda_{i,k}$. This changes the error covariance $P_{k}^{-}$ and enables real-time adjustment of the Kalman gain $K_{k}$, thereby enhancing the tracking capability. According to Equation (14), when the system state is stable, the SKF algorithm reduces to the EKF algorithm.

### 3.2. Fuzzy-Controlled Forgetting-Factor Strong Tracking Kalman Filter

**Figure 2:**
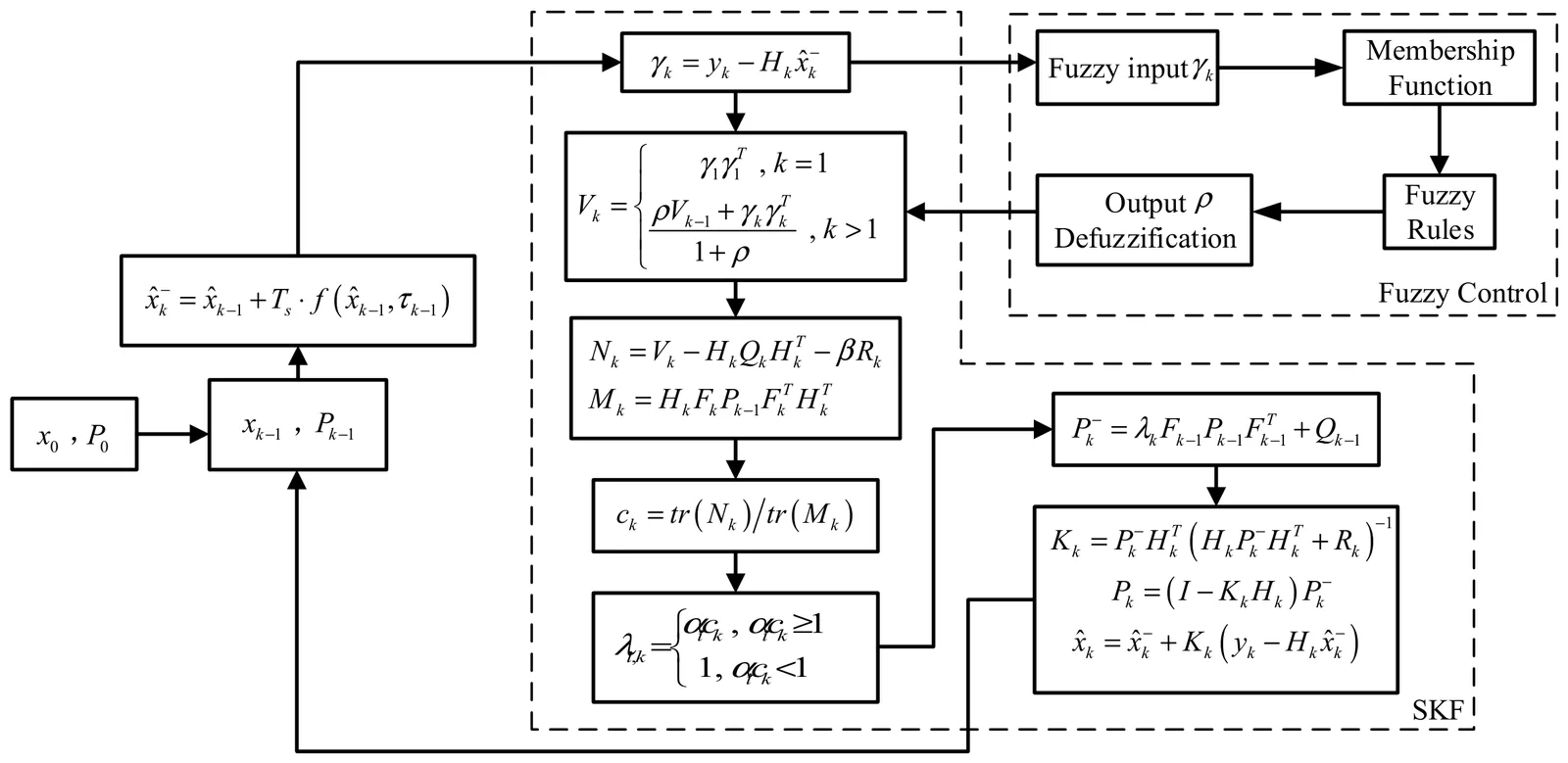
Fuzzy-controlled forgetting-factor SKF estimation.

In the SKF algorithm, the forgetting factor ρ is a fixed parameter and therefore must be estimated in advance according to simulation results or practical experience, making online adaptive parameter adjustment impossible [6]. To address this problem in the SKF algorithm, this paper combines fuzzy-control-based adjustment of the forgetting factor ρ with the SKF algorithm. The algorithm is as follows:

As shown in Figure 2, the FSKF algorithm mainly uses the input estimation error $\gamma_{k}$ as the input feature. It fuzzifies the mean value of $\gamma_{k}$, performs fuzzy inference using the rule base, and defuzzifies to adaptively obtain the adjusted forgetting factor ρ. Unlike the SKF algorithm, the FSKF algorithm realizes online adjustment of the forgetting factor ρ, thereby indirectly adjusting the fading factor $\lambda_{i,k}$. The fuzzy control design in the FSKF algorithm is as follows:

(1) Input and quantization of the estimation error $\gamma_{k}$.

The fuzzy input is quantified using the mean absolute values of the estimated position and velocity errors:

(19) $$\zeta =\frac{1}{n}\sum_{k=1}^{n}\left|\gamma_{k}\right|$$

(2) Fuzzification process.

**Figure 3:**
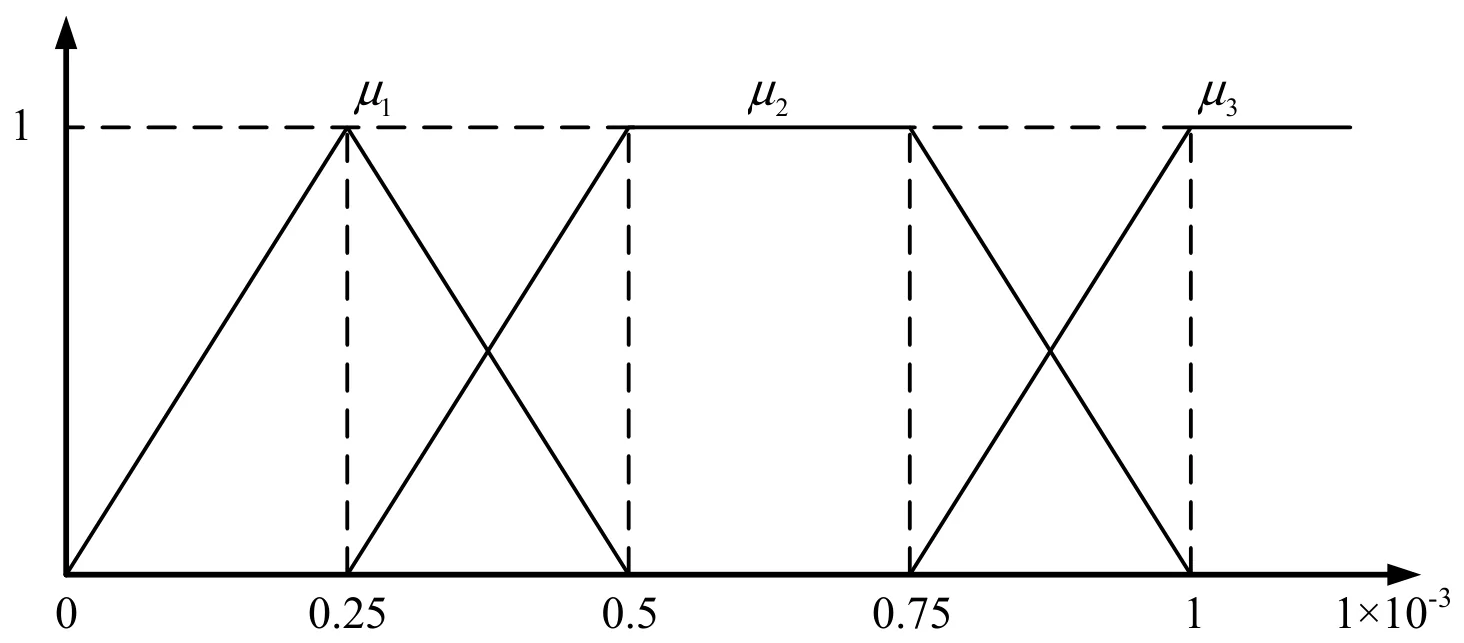
Membership functions.

The fuzzification process maps the input into fuzzy linguistic terms, which are represented by membership functions and expressed as linguistic variables. Common membership functions include triangular, trapezoidal, and Gaussian functions. In this paper, triangular and trapezoidal functions are selected as the membership functions. The fuzzy domain of the average estimation is defined as {0, 0.25, 0.5, 0.75, 1}, and the fuzzy subsets are defined as {zero, positive small, positive big}, denoted as {ZO, PS, PB}, respectively. Figure 3 shows the defined membership functions, and Equations (20)–(22) give their expressions.

(20) $$\mu_{1}=\left\{\begin{array}{cc} 4x & 0\leq x<0.25\\ -4x+2 & 0.25\leq x<0.5\\ 0 & others \end{array}\right.$$

(21) $$\mu_{3}=\left\{\begin{array}{cc} 4x-3 & 0.75\leq x<1\\ 1 & 1\leq x\\ 0 & others \end{array}\right.$$

(22) $$\mu_{2}=\left\{\begin{array}{cc} 4x-1 & 0.25\leq x<0.5\\ 1 & 0.5\leq x<0.75\\ -4x+4 & 0.75\leq x<1\\ 0 & others \end{array}\right.$$

(3) Definition of fuzzy rules.

The fuzzy logic system is a single-input, single-output system. Therefore the fuzzy rule library is represented as a one-dimensional mapping. The input is the average absolute value of the estimated position and velocity errors, and the output is the adaptively adjusted forgetting factor. The fuzzy inference rules can be obtained from the simulation and experimental results, and the fuzzy rules are shown in Table 2.

(4) Defuzzification.

Defuzzification calculates the output used for control according to the results of fuzzy inference. In this paper, the centroid method is adopted because it preserves the information contained in fuzzy rules. It is expressed as

(23) $$\rho =\frac{\sum_{i=1}^{n}\mu \left(\gamma_{i}\right)\rho_{i}}{\sum_{i=1}^{n}\mu \left(\gamma_{i}\right)}$$

Accordingly, the estimated contact force can be obtained from the above Kalman filtering algorithm as follows:

(24) $${\hat{F}}_{ext}=J^{-1}{\hat{\tau }}_{ext}$$

where ${\hat{F}}_{ext}$ is the estimated contact force, and J is the Jacobian matrix.

## 4. Adaptive Variable-Damping Impedance Control Based on Contact Force Estimation

To improve the adaptability of the robotic arm to the environment, impedance control is generally introduced to provide compliant behavior. However, when a conventional impedance controller faces an unknown environment, accurate force tracking is difficult to achieve [5,21]. Therefore, an adaptive impedance controller is proposed. By adjusting the damping coefficient, it compensates for errors caused by uncertainty and provides fast tracking performance.

### 4.1. Contact Force and Impedance Control Model

**Figure 4:**
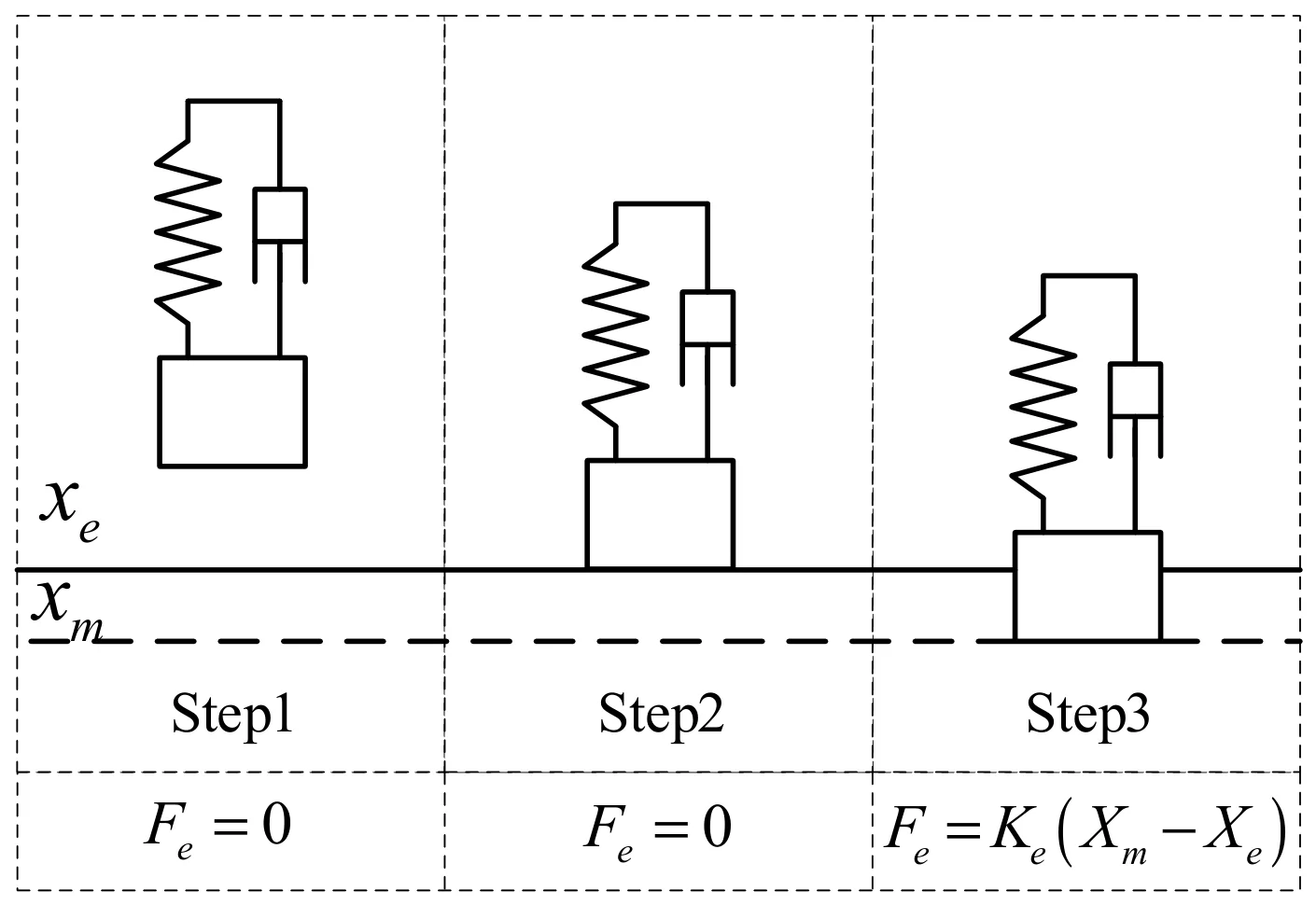
Contact force model.

The generation of contact force between the robotic arm and the environment consists of three stages, as shown in Figure 4. In the first stage, the robot is not in contact with the environment, $F_{e}=0$; in the second stage, the robot contacts the environment but the environment is not deformed, $F_{e}=0$; in the third stage, the robot contacts the environment and deformation occurs, $F_{e}=K_{e}\left(X_{m}-X_{e}\right)$.

**Figure 5:**
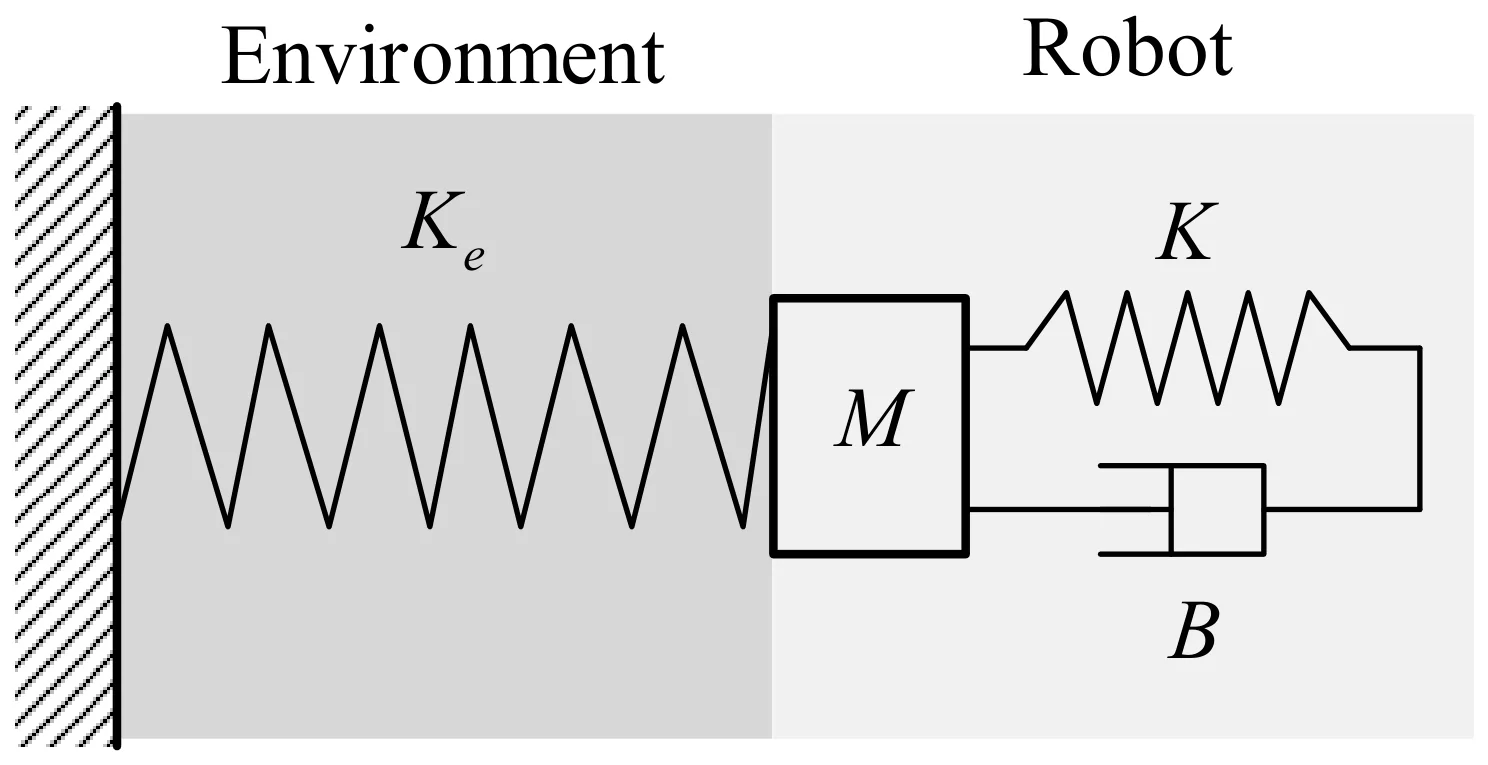
Robot impedance model.

The robot impedance model is shown in Figure 5. The environment is modeled as a linear spring. When the robot contacts the environment and deformation occurs, the contact force is

(25) $$F_{e}=K_{e}\left(X_{m}-X_{e}\right)$$

where $F_{e}$ is the contact force, $K_{e}$ is the environmental stiffness, $X_{e}$ is the environmental position, and $X_{m}$ is the robot end-effector position.

Impedance control treats the robotic arm as an equivalent second-order differential system consisting of mass, damping, and stiffness. When it is subjected to a contact force $F_{e}$, it produces an action that counteracts the contact force, so that the resultant force at the contact surface is $F_{d}$. The expression is as follows:

(26) $$M\ddot{E}+B\dot{E}+KE=F_{d}-F_{e}=\Delta F$$

where M, B, and K are the inertia, damping, and stiffness coefficients, respectively; $E=X_{m}-X_{e}$ is the deformation of the environment. Therefore, the transfer model of impedance control is

(27) $$G\left(s\right)=\frac{E\left(s\right)}{F\left(s\right)}=\frac{1}{\left(Ms^{2}+Bs+K\right)}$$

**Figure 6:**
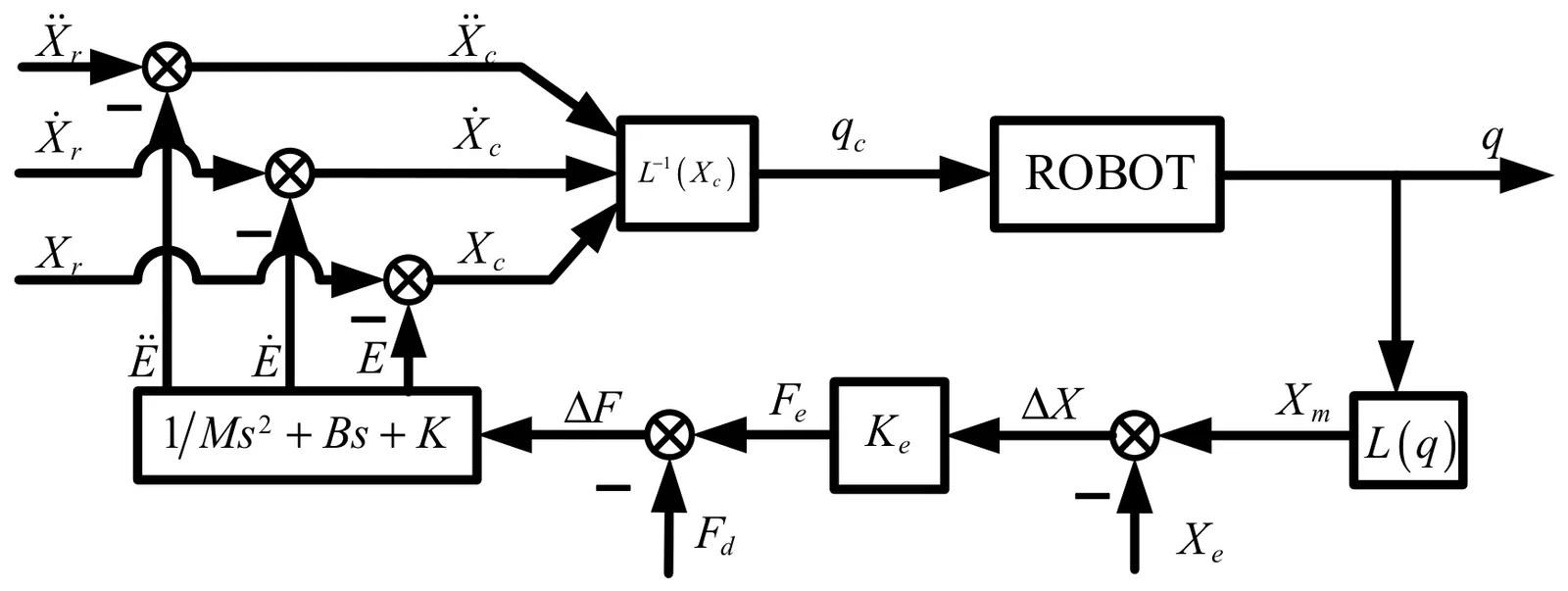
Block diagram of position-based impedance control.

The block diagram of position-based impedance control is shown in Figure 6. When the robotic arm contacts the environment and generates a contact force $F_{e}$, to achieve the desired force $F_{d}$, the force error ΔF is used under impedance control to obtain the correction $E=\Delta F\cdot G\left(s\right)$, which corrects the reference trajectory $X_{r}$ and obtains the control trajectory $X_{c}$, so that the end effector moves from $X_{m}$ to $X_{c}$.

Assuming that the robot performs force tracking in a single dimension, the corrected control trajectory is

(28) $$x_{c}=x_{r}+e$$

Substituting Equation (28) into Equation (27), the force tracking error Δf is obtained as:

(29) $$\Delta f=f_{d}-f_{e}=f_{d}-k_{e}\left(x_{e}-x_{c}\right)$$

Substituting Equation (27) into Equation (29) and rearranging yields

(30) $$\Delta f\left(ms^{2}+bs+k+k_{e}\right)=\left(ms^{2}+bs+k\right)\left[f_{d}-k_{e}\left(x_{e}-x_{r}\right)\right]$$

The force tracking error is $\Delta f_{ss}$:

(31) $$\Delta f_{ss}=\frac{k}{k+k_{e}}\left[f_{d}-k_{e}\left(x_{e}-x_{r}\right)\right]$$

From Equation (31), to make $\Delta f_{ss}=0$, the following conditions must be satisfied:

(32) $$x_{e}=x_{r}+\frac{f_{d}}{k_{e}}$$

As shown in Equation (32), if the environment position and stiffness are known in advance, a position trajectory that makes the force tracking error zero can be calculated beforehand. In practice, however, the true environment position and stiffness cannot be known accurately in advance, making it difficult to calculate a position trajectory that makes the force-tracking error zero. Therefore, a stable force tracking error will exist. Hence, it is particularly important to design an adaptive impedance controller to address this problem.

### 4.2. Adaptive Variable-Damping Impedance Control

To achieve force tracking by the robotic arm, an adaptive variable-damping impedance control strategy is proposed. It is assumed that, in the position servo mode, the end-effector trajectory of the robotic arm always reaches the control trajectory. Therefore, the impedance control equation is

(33) $$\Delta f=m\ddot{e}+b\dot{e}+ke$$

where $e=x_{m}-x_{e}=x_{c}-x_{e}$.

In free space, when $f_{e}=0$, the impedance control becomes

(34) $$m\ddot{e}+b\dot{e}+ke=f_{d}$$

From Equation (34), when $f_{d}=0$, $x_{c}=x_{e}$ is satisfied, and the force tracking error is $\Delta f_{ss}=0$. During contact, if $f_{d}\neq 0$, that is, if a desired force is maintained at the end effector, then $x_{c}\neq x_{e}$. To make $\Delta f_{ss}=0$, the stiffness coefficient must satisfy k=0, such that, for any environmental stiffness $k_{e}$, the system has $f_{e}=f_{d}$ at steady state.

For a planar environment, ${\dot{x}}_{e}={\ddot{x}}_{e}=0$, so $\Delta f=m{\ddot{x}}_{c}+b{\dot{x}}_{c}$ and $\Delta f_{ss}=0$ exist; for an uncertain surface, ${\dot{x}}_{e}\neq 0$ and ${\ddot{x}}_{e}\neq 0$, and therefore the environment parameters need to be estimated. Assuming that the estimated environment parameter is ${\hat{x}}_{e}=x_{e}-\delta x_{e}$ and the trajectory error is $\hat{e}=e+\delta x_{e}$. Substituting the trajectory error $\hat{e}$ into the impedance model, the adaptive variable damping coefficient $\Delta b\left(t\right)$ is defined as follows:

(35) $$\Delta f=m\ddot{\hat{e}}+b\dot{\hat{e}}+\Delta b\left(t\right)\dot{\hat{e}}$$

Considering the force error $e_{f}=f_{d}-f_{e}$ and its rate of change ${\dot{e}}_{f}$, $\Delta b\left(t\right)$ can be designed as

(36) $$\Delta b\left(t\right)=\left(k_{u}e_{f}-k_{v}{\dot{e}}_{f}\right)b/\varepsilon$$

where $k_{u}$ and $k_{v}$ are gain coefficients; ε is used to prevent the denominator from becoming zero and satisfies

$$\varepsilon =\left\{\begin{array}{cc} \dot{\hat{e}} & \left|\dot{\hat{e}}\right|>10^{-8}\\ 10^{-8} & otherwise \end{array}\right.$$

**Figure 7:**
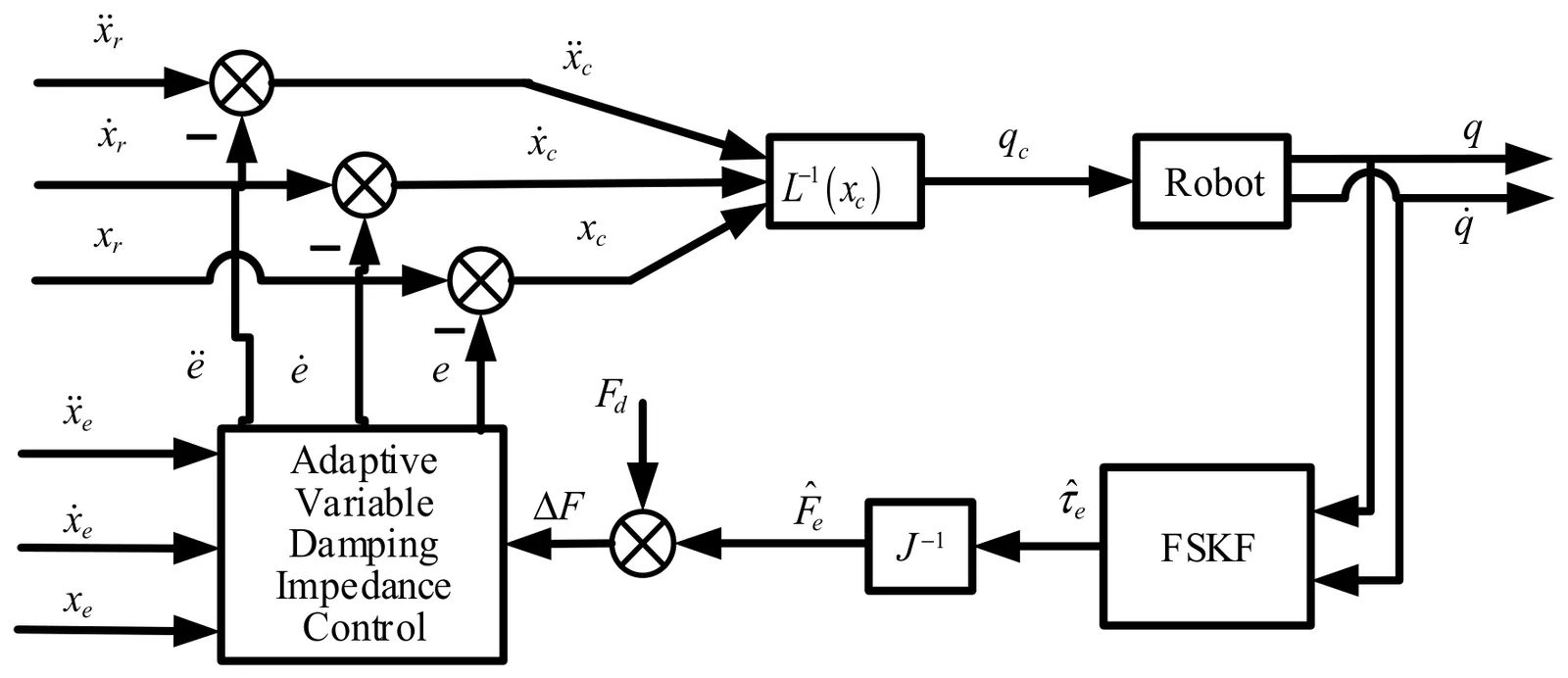
Control block diagram of the robotic arm.

According to the external torque estimated by the FSKF, the contact force $F_{e}$ is replaced by the estimated contact force ${\hat{F}}_{e}$. The control block diagram of the robotic arm is shown in Figure 7. The inner control loop of the robotic arm is a position-control loop, whereas the outer control loop is an adaptive variable-damping impedance-control loop.

### 4.3. Stability Analysis

To prove the stability of the variable-damping impedance controller, the stability of the closed-loop system needs to be established. Assume that $k_{n}$ is positive, and define the Lyapunov function as

(37) $$V=\frac{1}{2}m{\dot{\hat{e}}}^{2}+\frac{1}{2}k_{n}e_{f}^{2}$$

Substituting the force tracking error $e_{f}$ into Equation (37) gives

(38) $$e_{f}=-m\ddot{\hat{e}}-b\dot{\hat{e}}-\Delta b\left(t\right)\dot{\hat{e}}=-m\ddot{\hat{e}}-b\dot{\hat{e}}-k_{u}^{\prime }\mu e_{f}+k_{v}^{\prime }\mu {\dot{e}}_{f}$$

where $k_{u}^{\prime }=bk_{u}$, $k_{v}^{\prime }=bk_{v}$, and $\mu =\dot{\hat{e}}/\varepsilon$.

Equation (38) can be written as

(39) $$m\ddot{\hat{e}}=-b\dot{\hat{e}}-\left(k_{u}^{\prime }\mu +1\right)e_{f}+k_{v}^{\prime }\mu {\dot{e}}_{f}$$

Assuming that the contact force is constant, the following can be obtained from the environmental contact force model:

(40) $$e_{f}=f_{d}-f_{e}=f_{d}-k_{e}\hat{e}$$

(41) $${\dot{e}}_{f}=-{\dot{f}}_{e}=-k_{e}\dot{\hat{e}}$$

Taking the derivative of the Lyapunov function in Equation (37) gives

(42) $$\dot{V}=m\dot{\hat{e}}\ddot{\hat{e}}+k_{n}e_{f}{\dot{e}}_{f}$$

Substituting Equations (39)–(41) into Equation (42) gives

(43) $$\dot{V}=-\left(b+\mu k_{v}^{\prime }k_{e}\right){\dot{\hat{e}}}^{2}-\left(\mu k_{u}^{\prime }+k_{n}k_{e}+1\right)e_{f}\dot{\hat{e}}$$

When $\varepsilon =\dot{\hat{e}}$, define $k_{n}=-\left(k_{u}^{\prime }+1\right)/k_{e}$; then

(44) $$\dot{V}=-\left(b+k_{v}^{\prime }k_{e}\right){\dot{\hat{e}}}^{2}\leq 0$$

When $\varepsilon =10^{-8}$, define $k_{n}=-k_{e}^{-1}$; then

(45) $$\dot{V}=-b{\dot{\hat{e}}}^{2}-\left(k_{n}k_{e}+1\right)e_{f}\dot{\hat{e}}-\mu k_{u}^{\prime }e_{f}\dot{\hat{e}}-\mu k_{v}^{\prime }k_{e}{\dot{\hat{e}}}^{2}\leq -b{\dot{\hat{e}}}^{2}-\left(k_{n}k_{e}+1\right)e_{f}\dot{\hat{e}}\leq 0$$

The derivative of the Lyapunov function is negative semidefinite, demonstrating the stability of the force-tracking scheme based on the adaptive variable-damping impedance controller.

## 5. Simulation and Experiment

### 5.1. Simulation

**Figure 8:**
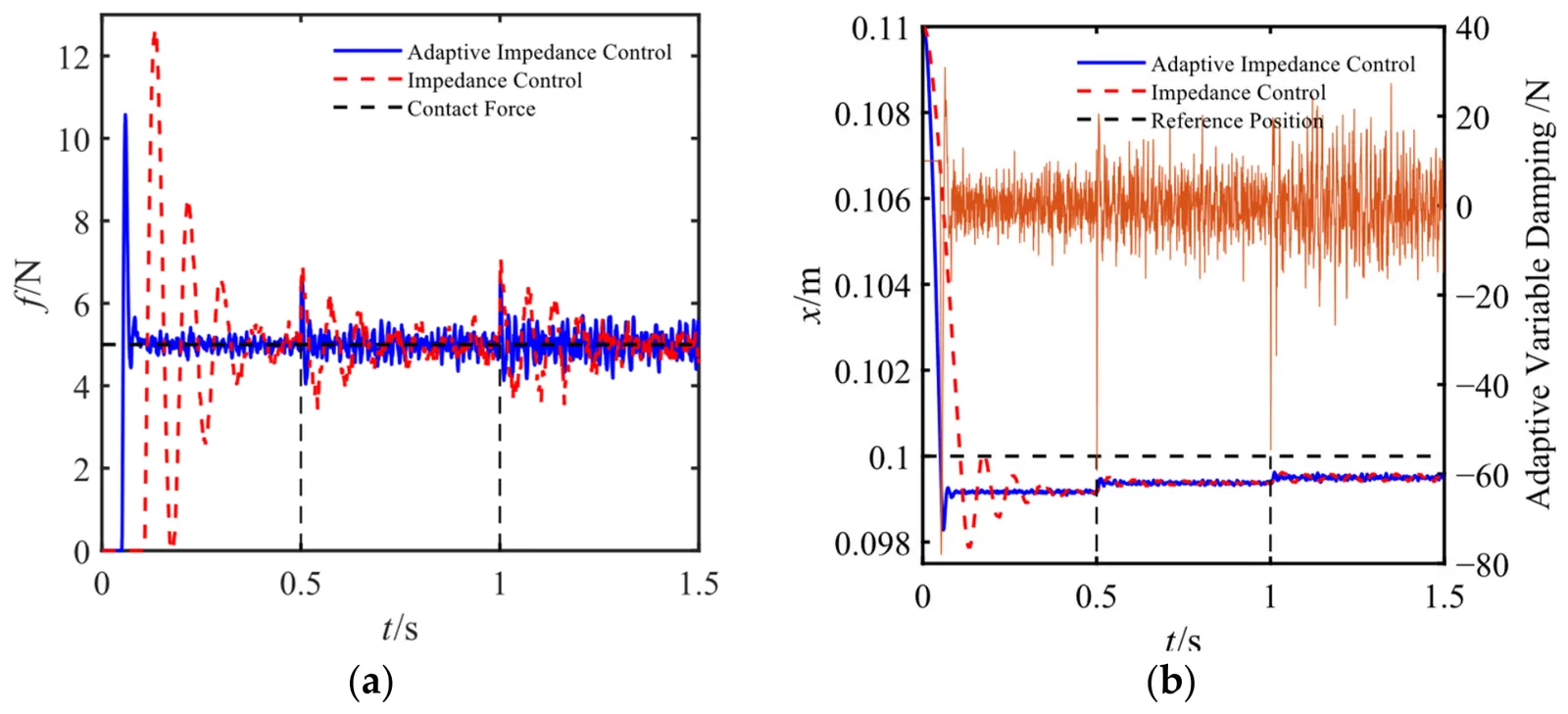
Simulation results of planar motion of 7-DOF biomimetic robotic arm: (**a**) Planar force tracking comparison. (**b**) Planar trajectory tracking comparison.

**Figure 9:**
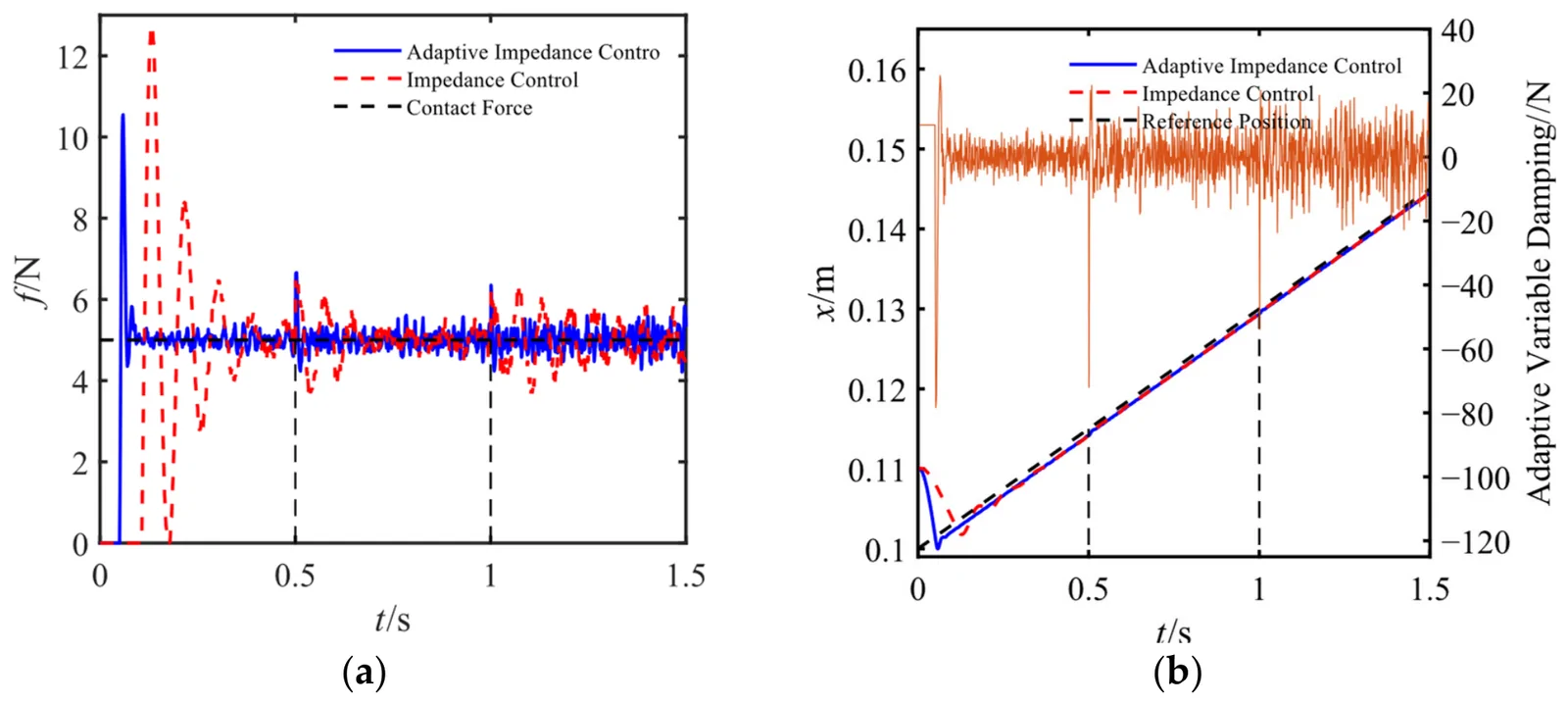
Simulation results of 7-DOF biomimetic robotic arm moving along the inclined plane: (**a**) Inclined-plane force tracking comparison. (**b**) Inclined-plane trajectory tracking comparison.

**Figure 10:**
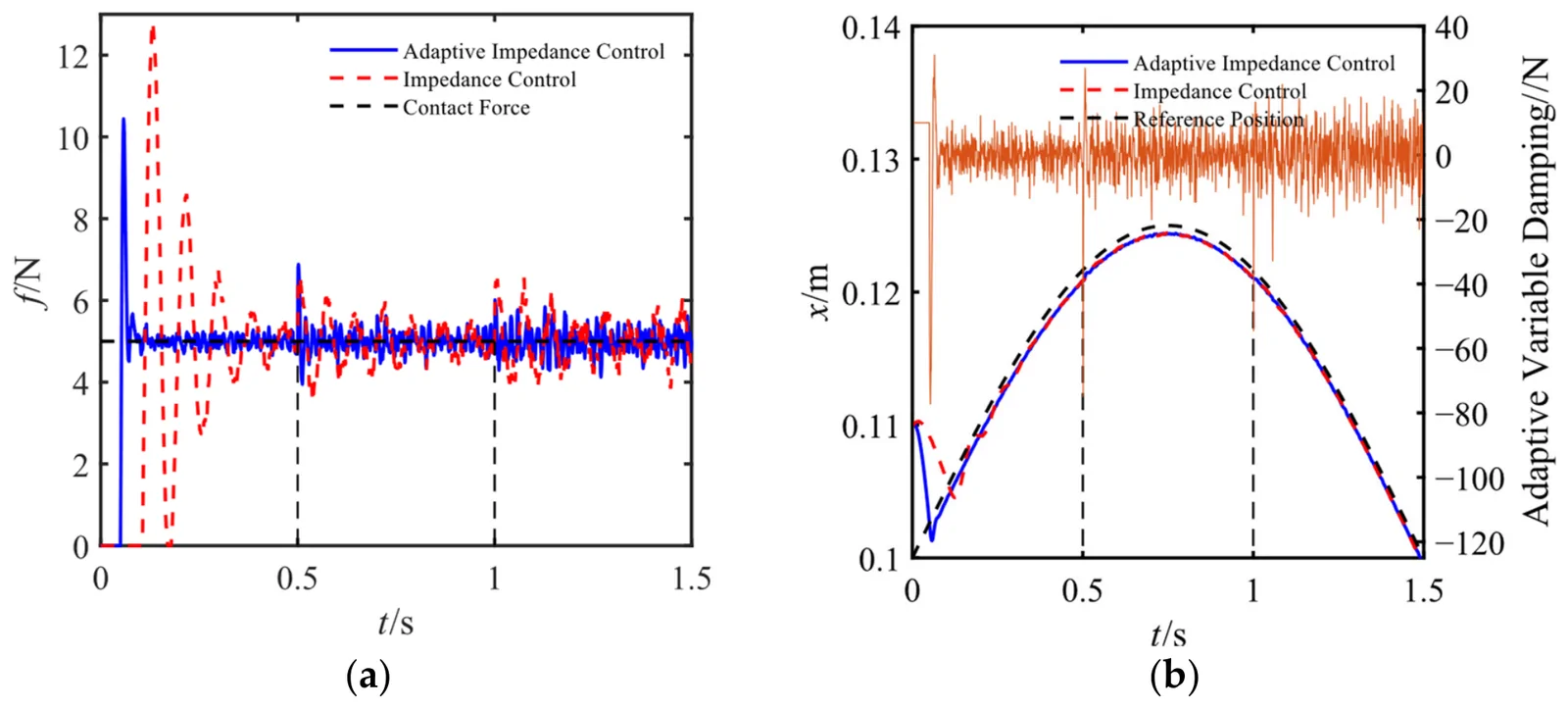
Simulation results of 7-DOF biomimetic robotic arm moving along the sinusoidal curved surface: (**a**) Sinusoidal curved surface force tracking comparison. (**b**) Sinusoidal curved surface trajectory tracking comparison.

To verify tracking as the robotic arm moves from free space to constrained space, a series of simulations is conducted to compare the force and position tracking of impedance control and adaptive variable-damping impedance control on contact surfaces in different environments. Figure 8, Figure 9 and Figure 10a show the force tracking comparisons between impedance control and adaptive variable-damping impedance control. Figure 8, Figure 9 and Figure 10b show the trajectory tracking comparisons of impedance control and adaptive variable-damping impedance control and the variation in the variable damping term in adaptive impedance control. The desired force is set as $f_{d}=5N$, the initial position of the robotic arm as $x_{c}=0.11m$, the impedance controller parameters as m=1 and b=40, and the adaptive control parameters as $k_{u}=0.05$ and $k_{v}=0.001$. White noise is added to each joint angle to simulate actual measurement conditions. The planar environment is set as $x_{e}=0.1m$; the inclined-plane environment is $x_{e}=0.05t+0.1m$; the sinusoidal environment is $x_{e}=0.025\sin\left[\left(2\pi /3\right)t\right]+0.1m$; and the environmental stiffness varies with time as

$$k_{e}=\left\{\begin{array}{cc} 6000 & 0\leq t<0.5\\ 8000 & 0.5\leq t<1\\ 10000 & 1\leq t\leq 1.5 \end{array}\right.$$

As shown in Figure 8, Figure 9 and Figure 10a, when the robotic arm moves from free space into contact with the environment, the contact force exhibits oscillations and overshoot. At 0.5 s and 1 s, the force tracking response of the robotic arm also exhibits slight oscillation and overshoot. However, under adaptive variable-damping impedance control, the robotic arm has smaller overshoot and faster response. Figure 8, Figure 9 and Figure 10b show that both algorithms enable stable trajectory tracking. After contact occurs, adaptive variable-damping impedance control responds quickly. The high-frequency oscillation curve in Figure 8, Figure 9 and Figure 10b is the variable damping term. When the environmental stiffness changes, the damping term rapidly decreases, making the robotic arm more compliant so that it can adapt to position changes caused by environmental variation. Compared with conventional impedance control, the adaptive variable-damping impedance controller exhibits better dynamic response and disturbance-rejection capability. Therefore, the adaptive variable-damping impedance controller shows better performance and higher disturbance-rejection capability.

**Figure 11:**
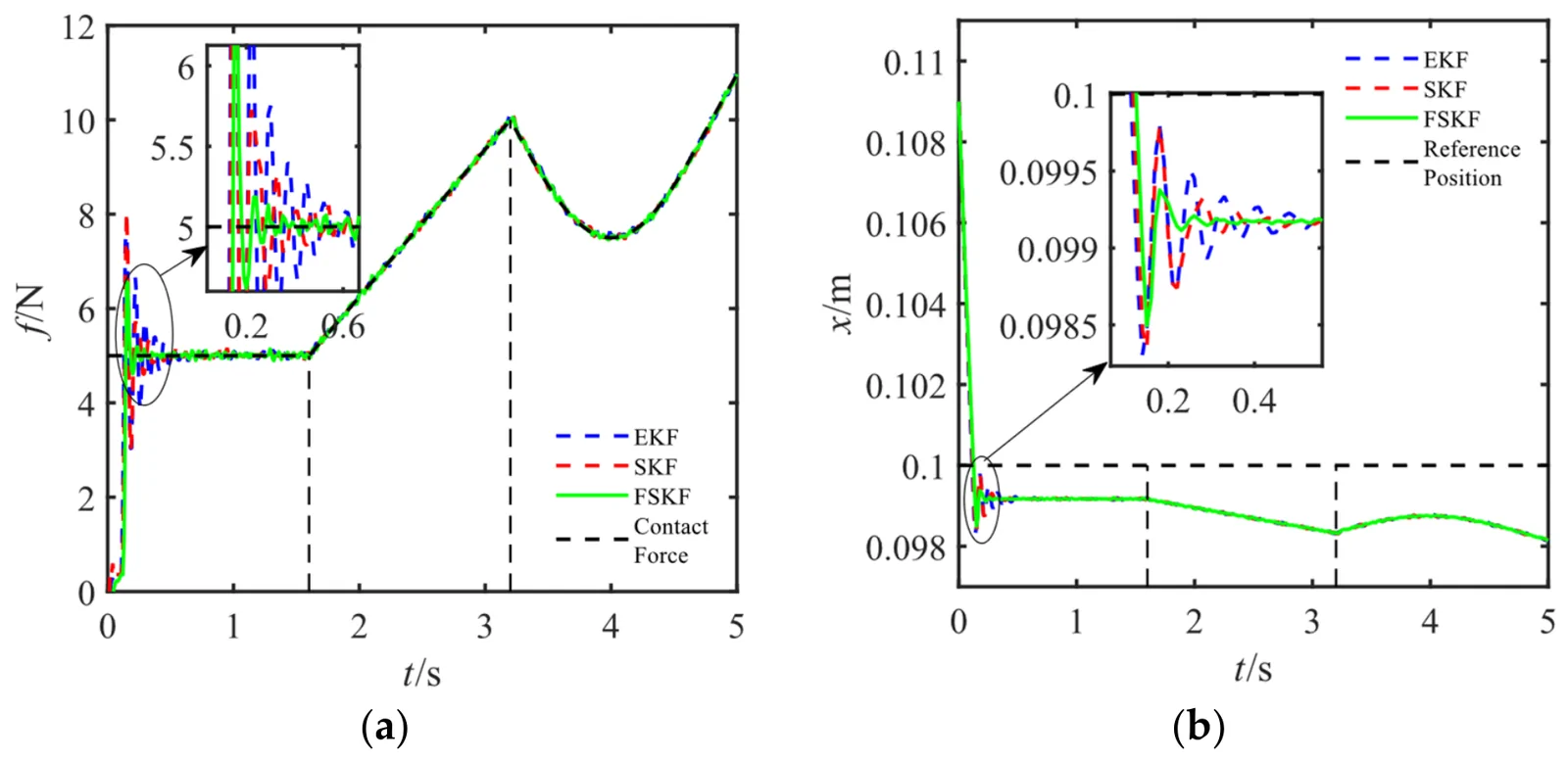
Comprehensive simulation of planar motion: (**a**) Planar force tracking comparison. (**b**) Planar trajectory tracking comparison.

**Figure 12:**
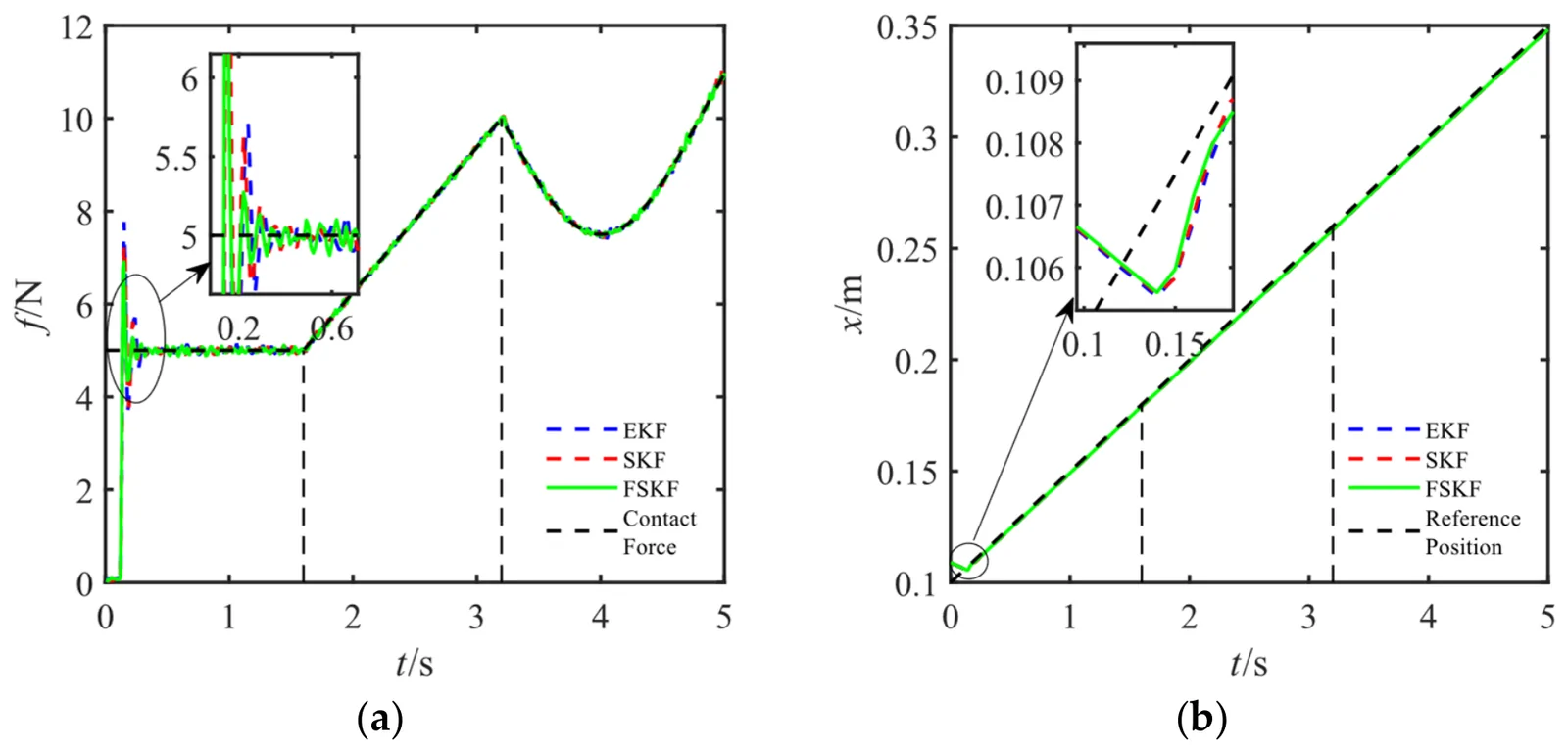
Comprehensive simulation of inclined-plane motion: (**a**) Planar force tracking comparison. (**b**) Planar trajectory tracking comparison.

**Figure 13:**
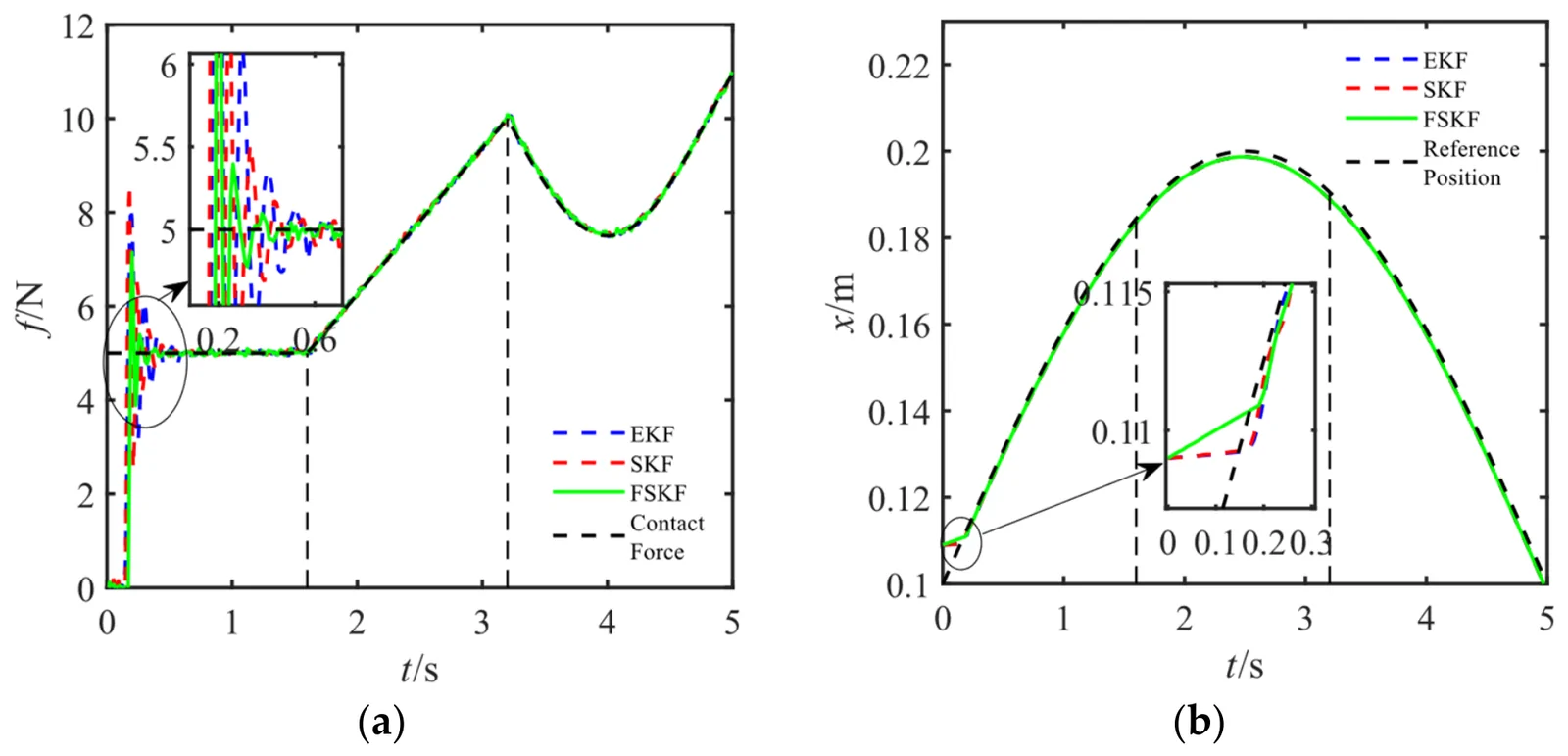
Comprehensive simulation of motion along the sinusoidal curved surface: (**a**) Planar force tracking comparison. (**b**) Planar trajectory tracking comparison.

Considering that adaptive variable-damping impedance control must adapt to changes in environmental conditions, two cases are considered in which the robot is subject to changes in the environment position and the desired contact force. The contact forces estimated by three Kalman filtering algorithms are used as feedback in the adaptive variable-damping impedance controller for simulation, and the contact force estimation performance of the three Kalman filtering algorithms is compared and verified. Figure 11, Figure 12 and Figure 13a show the comparisons of the contact force estimates obtained by the three algorithms, and Figure 11, Figure 12 and Figure 13b show the trajectory tracking comparisons of the three algorithms. The initial position of the robotic arm is set as $x_{c}=0.11m$; the impedance controller parameters are m=1 and b=85; the planar environment is $x_{e}=0.1m$; the inclined-plane environment is $x_{e}=0.1+0.05t\;m$; the sinusoidal curved-surface environment is $x_{e}=0.1\sin\left(0.2\pi t\right)+0.1m$; and the environmental stiffness is $k_{e}=6000$. The SKF algorithm parameters are set as ρ=0.95, β=1, and $\alpha =I_{21\times 21}$. The FSKF algorithm parameters are set as β=1 and $\alpha =I_{21\times 21}$, and ρ is determined according to the fuzzy algorithm. The basic Kalman filtering parameters are set as

$$R=diag\left[\begin{array}{cc} 10I_{7\times 7} & 5I_{7\times 7} \end{array}\right]\times 10^{-4};\;Q=diag\left[\begin{array}{ccc} 2I_{7\times 7} & I_{7\times 7} & I_{7\times 7} \end{array}\right]\times 10^{-2};\;P=I_{21\times 21}\times 10^{5}$$

The desired contact force is set as a step contact force, ramp contact force, and sinusoidal contact force at different times:

$$f_{d}=\left\{\begin{array}{cc} 5 & 0\leq t<1.6\\ 3.125t & 1.6\leq t<3.2\\ -2.5\sin\left(0.625\pi t\right)+10 & 3.2\leq t\leq 5 \end{array}\right.$$

As shown in Figure 11, Figure 12 and Figure 13a, when the contact force estimates of the three algorithms are fed back to the adaptive variable-damping impedance controller and the robotic arm moves from free space to constrained space, the contact force oscillates and then converges. FSKF provides better contact force estimation and achieves force tracking effectively. Figure 11, Figure 12 and Figure 13b show that the robotic arm can track the trajectory under all three algorithms, and FSKF provides better tracking capability at the initial stage. As the robotic arm operates steadily, FSKF and SKF degenerate into EKF, after which their contact force and trajectory tracking performances become similar. Table 3, Table 4 and Table 5 show the RMSE of force tracking and position tracking based on the simulation results. The simulation in which the estimated contact force is fed back to the adaptive variable-damping impedance control shows that the overall capability of FSKF is superior to that of SKF and EKF in terms of tracking performance.

### 5.2. Experiments

#### 5.2.1. Contact Force Estimation Experiment

**Figure 14:**
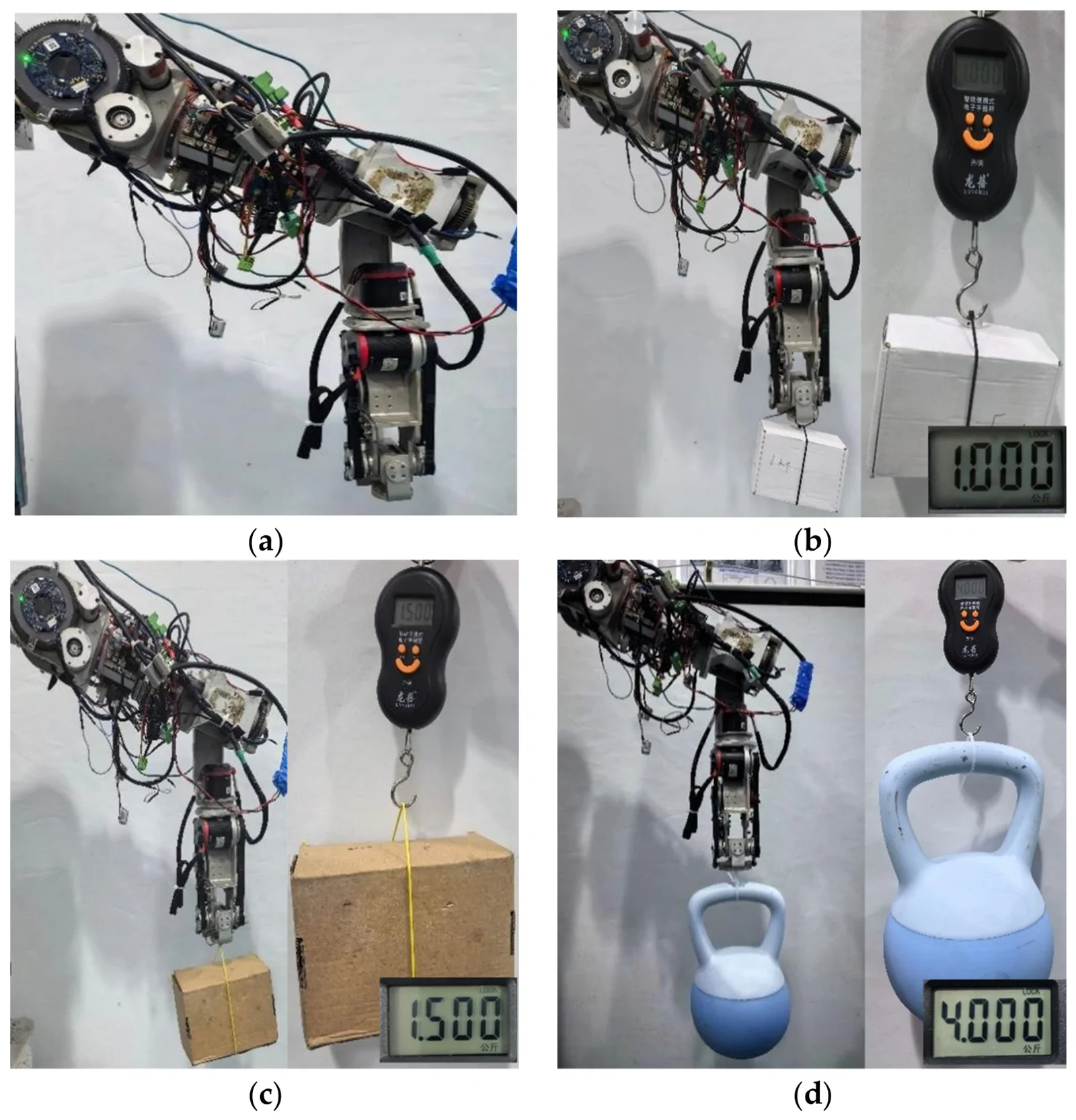
Experimental validation of the contact force estimation algorithms. All values are mass meter readings in kg.: (**a**) Prosthetic-arm end load of 0 kg. (**b**) Prosthetic-arm end load of 1.0 kg. (**c**) Prosthetic-arm end load of 1.5 kg. (**d**) Prosthetic-arm end load of 4.0 kg.

In the experiments, a 7-DOF biomimetic robotic arm was used as a representative biomimetic robotic-arm platform. External loads are applied to the end effector of the prosthetic arm, and the three algorithms are used to estimate the resulting contact force. Loads of 0 kg, 1.0 kg, 1.5 kg, and 4.0 kg are vertically applied to the end effector of the prosthetic arm, as shown in Figure 14. The joint angle, angular velocity, and input torque data provided by the prosthetic-arm motors are acquired through the host computer. While the loads are maintained in the vertical downward direction, the robotic arm is operated, and the acquired data are fed into the EKF, SKF, and FSKF algorithms to estimate the contact force.

**Figure 15:**
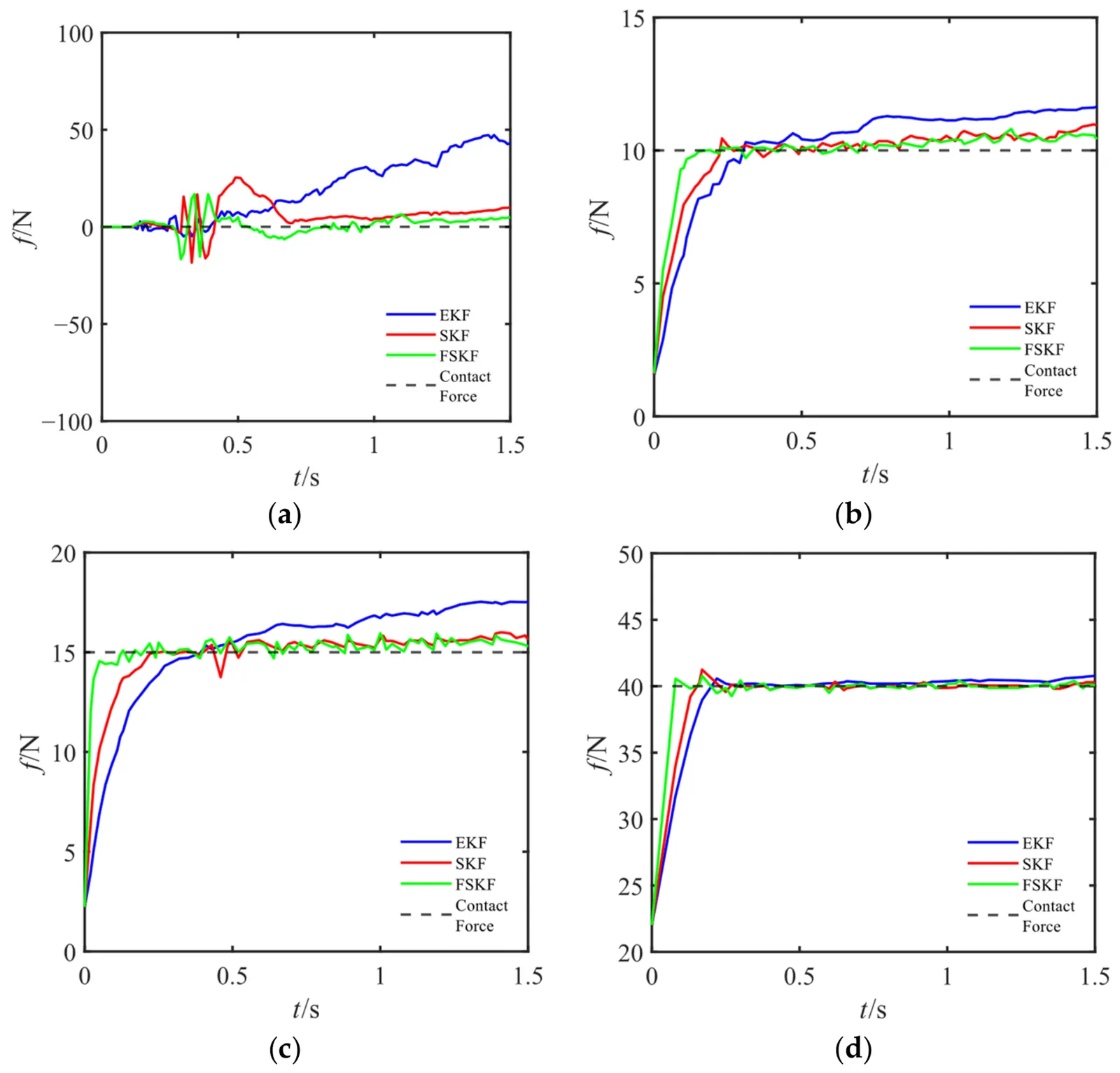
Estimates obtained by different contact force estimation algorithms: (a) Contact force estimate with a 0 kg load. (**b**) Contact force estimate with a 1 kg load. (**c**) Contact force estimate with a 1.5 kg load. (**d**) Contact force estimate with a 4 kg load.

Figure 15 shows the contact forces estimated by the different Kalman filtering algorithms when loads of 0 kg, 1.0 kg, 1.5 kg, and 4.0 kg are applied to the end effector of the 7-DOF prosthetic arm.

The results show that the FSKF provides higher estimation accuracy than the SKF and EKF, whereas the EKF cannot accurately estimate the contact force when there is no or low load at the end. This may be due to the limited accuracy of the motor sensors; the measured joint angles, angular velocities, and torque signals contain considerable noise, which degrades the estimation performance of the Kalman filtering algorithms. Consequently, the contact force cannot be accurately estimated during the later stage, and the estimation results exhibit relatively large fluctuations. During the initial estimation stage, the FSKF exhibits a faster convergence rate than the SKF, enabling it to track the desired force more rapidly. The experimental results obtained with the prosthetic arm demonstrate that the FSKF provides better contact force estimation performance than the EKF and SKF.

#### 5.2.2. Adaptive Impedance Control Experiment

**Figure 16:**
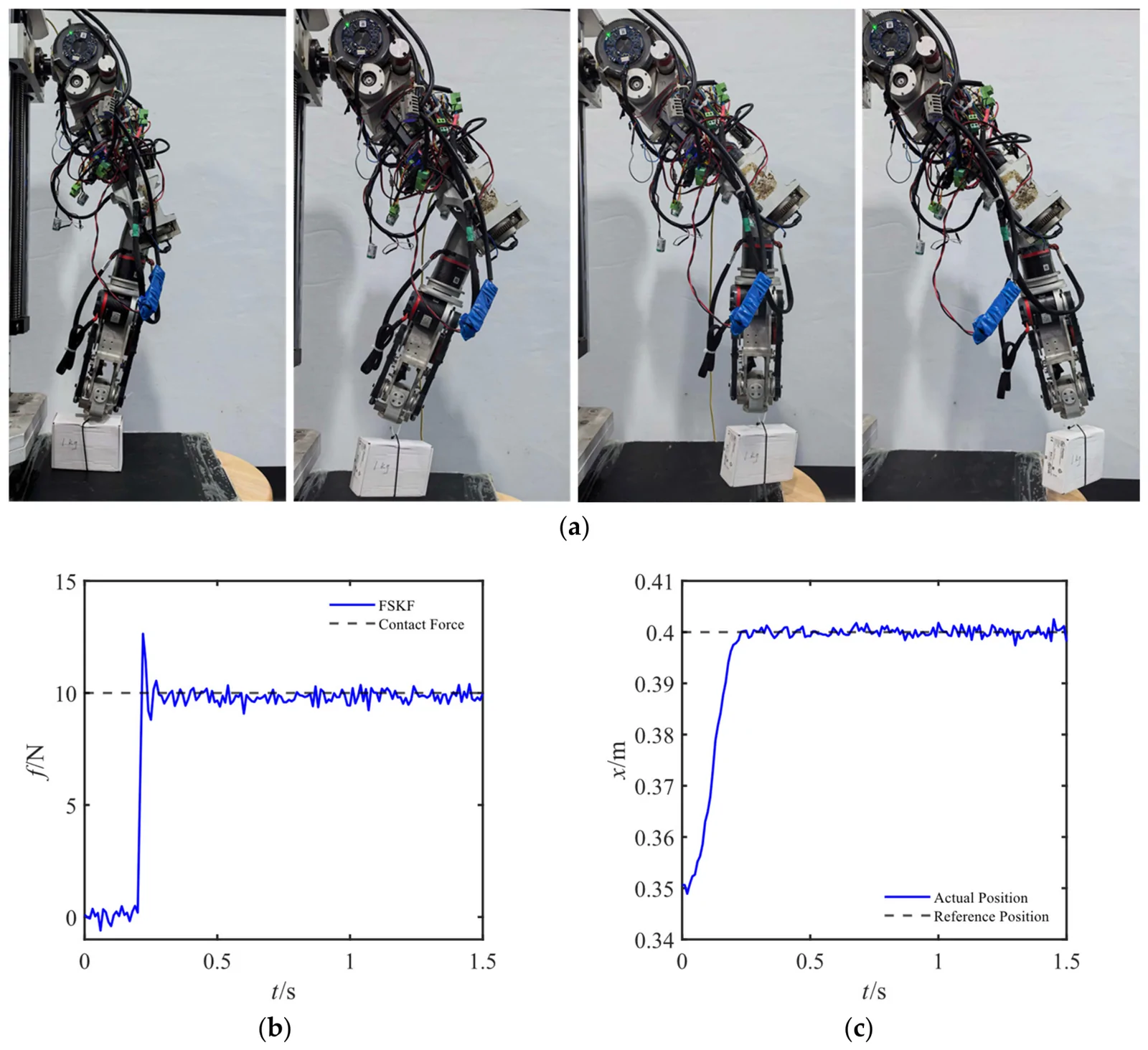
Adaptive impedance control experiment based on contact force estimation: (**a**) Robotic arm motion. (**b**) Contact force estimate. (**c**) End trajectory of the prosthetic arm.

The proposed adaptive variable-damping impedance control algorithm based on contact force estimation is experimentally validated. This experiment also verifies the force-tracking and position-tracking performance of the biomimetic robotic arm during a practical object-lifting task. The motion of the robotic arm is shown in Figure 16. Initially, no contact force is applied to the end effector. After the motion begins, the robotic arm lifts a 1 kg object attached to its end effector. The contact force is estimated using the FSKF, and the estimated force is fed back to the adaptive impedance controller for control calculation, thereby regulating the motion of the robotic arm. The robotic arm is assumed to move along a plane at a height of 0.4 m, and the desired contact force is set to the gravitational force acting on the 1 kg load.

As shown in Figure 16, when the desired contact force is 10 N, the adaptive variable-damping impedance controller achieves good force tracking, and the robotic arm can adjust its position while maintaining trajectory tracking. Before the weight is lifted, the estimated contact force fluctuates around zero due to system noise. After contact occurs, the robotic arm rapidly tracks the desired contact force and converges to it after slight oscillation and overshoot. Due to factors such as sensor accuracy, the end-effector trajectory exhibits noticeable fluctuations; however, the trajectory-tracking error of the robotic arm remains below 0.005 m. The adaptive variable-damping impedance control experiment demonstrates that the proposed method enables the robotic arm to achieve good compliant behavior while effectively performing force and trajectory tracking. The contact force estimated by the FSKF can be effectively used as force feedback for the adaptive impedance controller, providing accurate contact force estimation and further experimentally validating the feasibility of the proposed method.

## 6. Discussion

The simulation and experimental results demonstrate that combining FSKF-based contact force estimation with adaptive variable-damping impedance control improves the contact force estimation and trajectory-tracking accuracy of a 7-DOF biomimetic robotic arm. The fuzzy adjustment of the forgetting factor enables the proposed method to respond rapidly to sudden contact, explaining its superior initial performance compared with SKF and EKF. The simulation results further indicate that dynamically reducing the damping coefficient when environmental stiffness changes can enhance the compliance of the robotic arm. The accuracy of FSKF-based contact force estimation was approximately 7.3% higher than that of conventional methods. In the experiment, the controller tracked a 10 N target while maintaining the position error below 0.005 m, supporting its applicability to prosthetic systems without external force sensors. The present study has several limitations. The experiments were conducted on a single 7-DOF robotic platform and focused mainly on static payloads and predefined motion tasks. In addition, the proposed controller was not experimentally evaluated under human-in-the-loop interaction, varying payloads, or a broad range of environmental conditions. Therefore, further studies are required before the method can be considered for practical prosthetic deployment.

## 7. Conclusions

In this paper, the strong tracking Kalman filter algorithm is improved based on fuzzy control. The proposed method uses the state-estimation error as the fuzzy input and adaptively adjusts the forgetting factor, enabling the biomimetic robotic arm to rapidly estimate the contact force. In addition, the contact environment model of the biomimetic robotic arm is analyzed, and an adaptive variable-damping impedance controller is designed based on force tracking of the biomimetic robotic arm. This method dynamically adjusts the damping coefficient so that the robotic arm achieves good compliance. The simulation and experimental results show that the proposed method can effectively achieve force tracking and position tracking of the biomimetic robotic arm and can reduce the dependence on contact force sensors to a certain extent. The proposed method may contribute to the lightweight and economical design of prosthetic systems, although further validation under human-in-the-loop conditions is required, and the contact force estimated by the algorithm can also provide a basis for environmental interaction of a prosthetic hand. In future work, the prosthetic hand and prosthetic arm can be integrated to jointly investigate compliant force interaction in prosthetic systems.

## Figures and Tables

**Table 1:** DH parameters.

Link	*α*_*i*−1_	*a*_*i*−1_	*θ_i_*	*d_i_*
1	0°	0	0°	0
2	90°	0	−90°	0
3	90°	0	−90°	d_3_ = 313 mm
4	90°	0	−180°	0
5	90°	0	−180°	d_5_ = 313 mm
6	90°	0	−90°	0
7	−90°	0	−0°	0

**Table 2:** Fuzzy inference rules for adaptive forgetting-factor adjustment.

*ζ*	*ρ*
ZO	1
PS	0.55
PB	0.05

**Table 3:** Trajectory and force tracking RMSE in planar environment.

Expected Signal Strength	Step Expectation Force		Expected Force of Slope		Sinusoidal Expected Force	
RMSE	Force tracking	Trajectory tracking	Force tracking	Trajectory tracking	Force tracking	Trajectory tracking
EKF	0.5481	1.284 × 10^−4^	0.4052	8.647 × 10^−5^	0.3719	9.264 × 10^−5^
SKF	0.4356	0.763 × 10^−4^	0.3403	6.592 × 10^−5^	0.3505	7.068 × 10^−5^
FSKF	0.3904	0.684 × 10^−4^	0.3251	5.725 × 10^−5^	0.3062	6.019 × 10^−5^

**Table 4:** Trajectory and force tracking RMSE in sloping environment.

Expected Signal Strength	Step Expectation Force		Expected Force of Slope		Sinusoidal Expected Force	
RMSE	Force tracking	Trajectory tracking	Force tracking	Trajectory tracking	Force tracking	Trajectory tracking
EKF	0.5259	1.326 × 10^−4^	0.7825	9.005 × 10^−5^	0.3952	8.426 × 10^−5^
SKF	0.4083	0.703 × 10^−4^	0.6972	6.791 × 10^−5^	0.3719	6.149 × 10^−5^
FSKF	0.3751	0.689 × 10^−4^	0.6901	6.572 × 10^−5^	0.3406	6.095 × 10^−5^

**Table 5:** Trajectory and force tracking RMSE in sinusoidal surface environment.

Expected Signal Strength	Step Expectation Force		Expected Force of Slope		Sinusoidal Expected Force	
RMSE	Force tracking	Trajectory tracking	Force tracking	Trajectory tracking	Force tracking	Trajectory tracking
EKF	0.6058	1.332 × 10^−4^	0.7376	8.091 × 10^−5^	0.4078	8.635 × 10^−5^
SKF	0.5732	0.803 × 10^−4^	0.6359	7.526 × 10^−5^	0.3927	6.317 × 10^−5^
FSKF	0.5018	0.796 × 10^−4^	0.6207	6.358 × 10^−5^	0.3701	6.102 × 10^−5^

## Data Availability

The data and code of the current study can be obtained from the corresponding author upon reasonable request.

## Abbreviations

The following abbreviations are used in this manuscript:

DOF	Degree of Freedom
KF	Kalman Filter
EKF	Extended Kalman Filter
SKF	Strong Tracking Kalman Filter
FSKF	Fuzzy-Controlled Forgetting-Factor SKF Estimation
